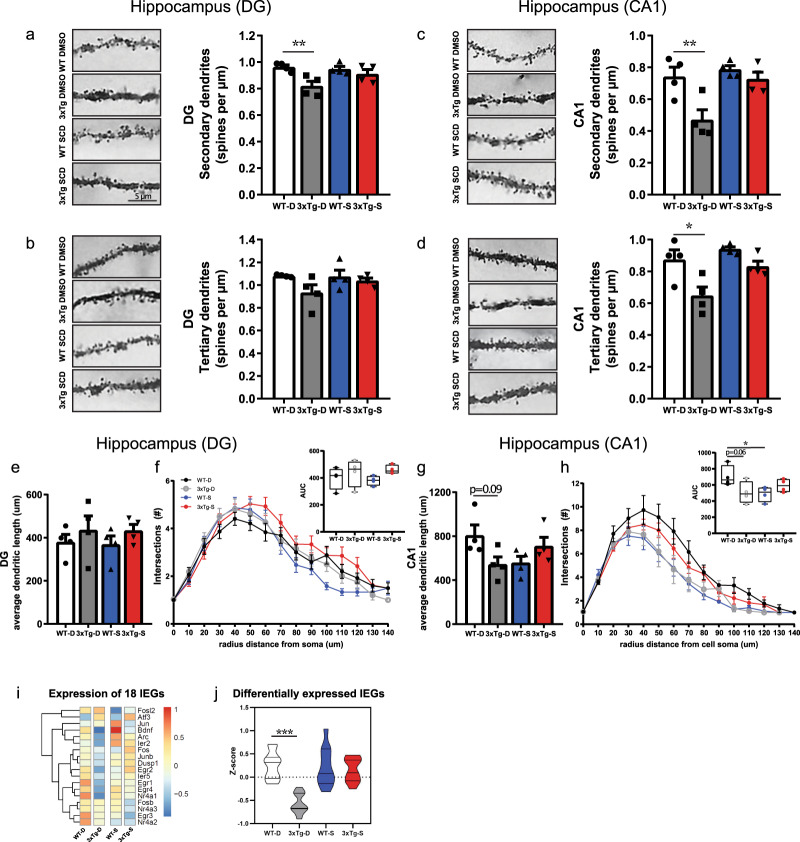# Author Correction: Stearoyl-CoA Desaturase inhibition reverses immune, synaptic and cognitive impairments in an Alzheimer’s disease mouse model

**DOI:** 10.1038/s41467-023-38295-x

**Published:** 2023-05-09

**Authors:** Laura K. Hamilton, Gaël Moquin-Beaudry, Chenicka L. Mangahas, Federico Pratesi, Myriam Aubin, Anne Aumont, Sandra E. Joppé, Alexandre Légiot, Annick Vachon, Mélanie Plourde, Catherine Mounier, Martine Tétreault, Karl J. L. Fernandes

**Affiliations:** 1 Research Center of the University of Montreal Hospital (CRCHUM), Université de Montréal, Montreal, QC Canada; 2 Department of Neurosciences, Faculty of Medicine, Université de Montréal, Montreal, QC Canada; 3 Research Center on Aging, CIUSSS de l’Estrie-CHUS, Sherbrooke, QC Canada; 4 Department of Medicine, Faculty of Medicine and Health Sciences, Université de Sherbrooke, Sherbrooke, QC Canada; 5 Department of Biological Sciences, Faculty of Science, Université de Québec à Montréal (UQAM), Montreal, QC Canada

**Keywords:** Neurology, Diseases of the nervous system

Correction to: *Nature Communications* 10.1038/s41467-022-29506-y, published online 20 April 2022

In the original version of the manuscript, the image shown in Figure 4c, third row down for the condition “WT SCD” was incorrect. This image incorrectly showed the same sample as shown in Figure 4a, top row for the condition “WT DMSO”. This has been corrected in the PDF and HTML versions of the article. The correct version of Figure 4 is as follows: