# Supplementary material for: Stearoyl-CoA Desaturase inhibition reverses immune, synaptic and cognitive impairments in an Alzheimer’s disease mouse model
Source: Nat Commun. 2022 Apr 20;13:2061. doi: 10.1038/s41467-022-29506-y (PMC9021296; doi:10.1038/s41467-022-29506-y)
Supplement: Supplementary file 1 — Supplemental Information [file 41467_2022_29506_MOESM1_ESM.pdf]

Supplemental Information

Supplemental Figure 1

a

Fatty acid (mg/g)	Strain and treatment				P-value
	WT-DMSO	WT-SCDi	3xTg-DMSO	3xTg-SCDi	
C14:0	0.045 ± 0.025	0.072 ± 0.033	0.051 ± 0.030	0.071 ± 0.016	ns
C15:0	0.012 ± 0.012	0.053 ± 0.009	0.035 ± 0.004	0.052 ± 0.006	ns
C16:0	7.179 ± 0.616	8.448 ± 0.957	6.494 ± 0.251	8.160 ± 1.043	WT_D vs WT_S : 0.007
					$3xTg_D$ vs $3xTg_S$: 0.001
C16:1 t	0.040 ± 0.021	0.055 ± 0.007	0.044 ± 0.004	0.069 ± 0.001	ns
C16:1	0.131 ± 0.022	0.140 ± 0.015	0.118 ± 0.018	0.147 ± 0.018	ns
C18:0	9.226 ± 0.743	9.955 ± 0.717	8.456 ± 0.291	9.469 ± 1.214	WT_D vs WT_S : 0.124
					WT_D vs $3xTg_D$: 0.129
					$3xTg_D$ vs $3xTg_S$: 0.046
C18:1 n-9	6.026 ± 0.912	5.888 ± 0.387	4.855 ± 0.770	5.683 ± 0.696	WT_D vs $3xTg_D$: 0.021
					$3xTg_D$ vs $3xTg_S$: 0.103
C18:1 n-7	1.400 ± 0.177	1.500 ± 0.114	1.247 ± 0.117	1.444 ± 0.234	ns
C18:2 t	0.573 ± 0.152	0.493 ± 0.061	0.416 ± 0.078	0.466 ± 0.061	ns
C18:2 n-6	0.290 ± 0.033	0.328 ± 0.027	0.303 ± 0.018	0.343 ± 0.053	ns
C20:0	0.104 ± 0.013	0.110 ± 0.007	0.122 ± 0.008	0.112 ± 0.006	ns
C20:1	0.559 ± 0.113	0.473 ± 0.056	0.511 ± 0.062	0.479 ± 0.024	ns
C20:3 n-6 DGLA	0.143 ± 0.023	0.147 ± 0.012	0.128 ± 0.013	0.155 ± 0.027	ns
C20:4 n-6	4.642 ± 0.498	4.976 ± 0.368	4.086 ± 0.476	4.958 ± 0.869	$3xTg_D$ vs $3xTg_S$: 0.086
C22:3	1.445 ± 0.235	1.479 ± 0.125	1.187 ± 0.161	1.393 ± 0.207	ns
C24:1	0.162 ± 0.085	0.076 ± 0.044	0.053 ± 0.053	0.000 ± 0.000	ns
C22:5 n-3 DPA	0.021 ± 0.021	0.043 ± 0.015	0.039 ± 0.020	0.048 ± 0.024	ns
C22:6 n-3 DHA	4.883 ± 0.550	5.209 ± 0.391	4.207 ± 0.544	5.489 ± 1.076	$3xTg_D$ vs $3xTg_S$: 0.012
∑SFA	20.31 ± 2.763	20.81 ± 1.490	17.19 ± 2.096	20.67 ± 3.283	WT_D vs $3xTg_D$: 0.124
					$3xTg_D$ vs $3xTg_S$: 0.087
∑UFA	16.57 ± 1.332	18.65 ± 1.659	15.16 ± 0.371	17.88 ± 2.259	ns
∑FA	36.88 ± 4.081	39.46 ± 2.828	32.35 ± 2.460	38.56 ± 5.527	WT_D vs $3xTg_D$: 0.027
					$3xTg_D$ vs $3xTg_S$: 0.003

b

Restored DEGs

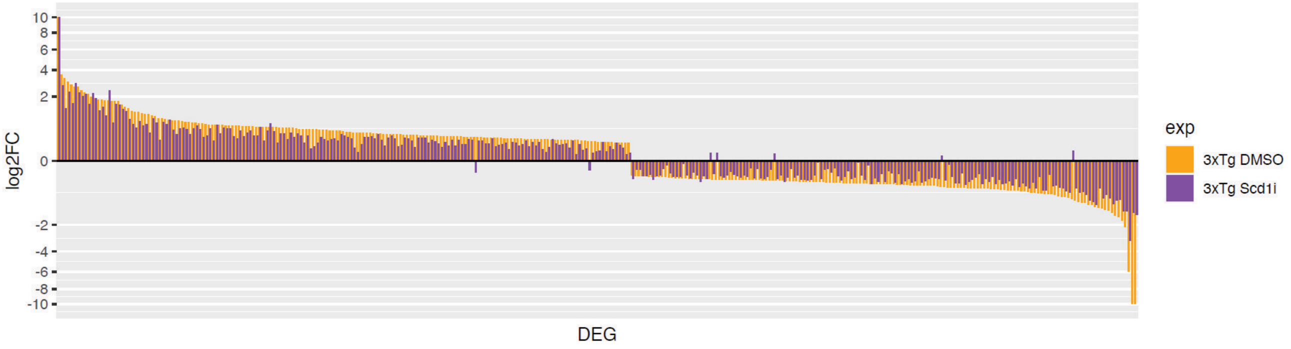

Supplemental Figure 1

a Results of quantitative GC-FID fatty acid profiling of the micro-dissected subventricular zone of DMSO- or SCDi-infused mice (N=3-4/group). Note that SCDi infusion in 3xTg mice led to increased concentrations (mg/g) of the SCD substrates C16:0 (palmitic acid, $p<0.001$) and C18:0 (stearic acid, $p=0.046$), as well as of C22:6 n-3 (DHA, $p=0.012$) and total fatty acids ($p=0.003$). Two-way ANOVA with uncorrected Fisher's LSD post-hoc test.

b Expression levels of the whole hippocampus "Restored" genes. Orange bars show the log2FC of the WT-D/3xTg-D DEGs that were "Restored". These are overlayed in purple with the log2FC of the same genes in the WT-D/3xTg-S comparison. Note that the difference in expression for the vast majority of WT-D/3xTg-D DEGs was reduced in the WT-D/3xTg-S comparison (an average 42% reduction in difference).

Related to **Figure 2**. Source data are provided as a Source Data file.

Supplemental Figure 2

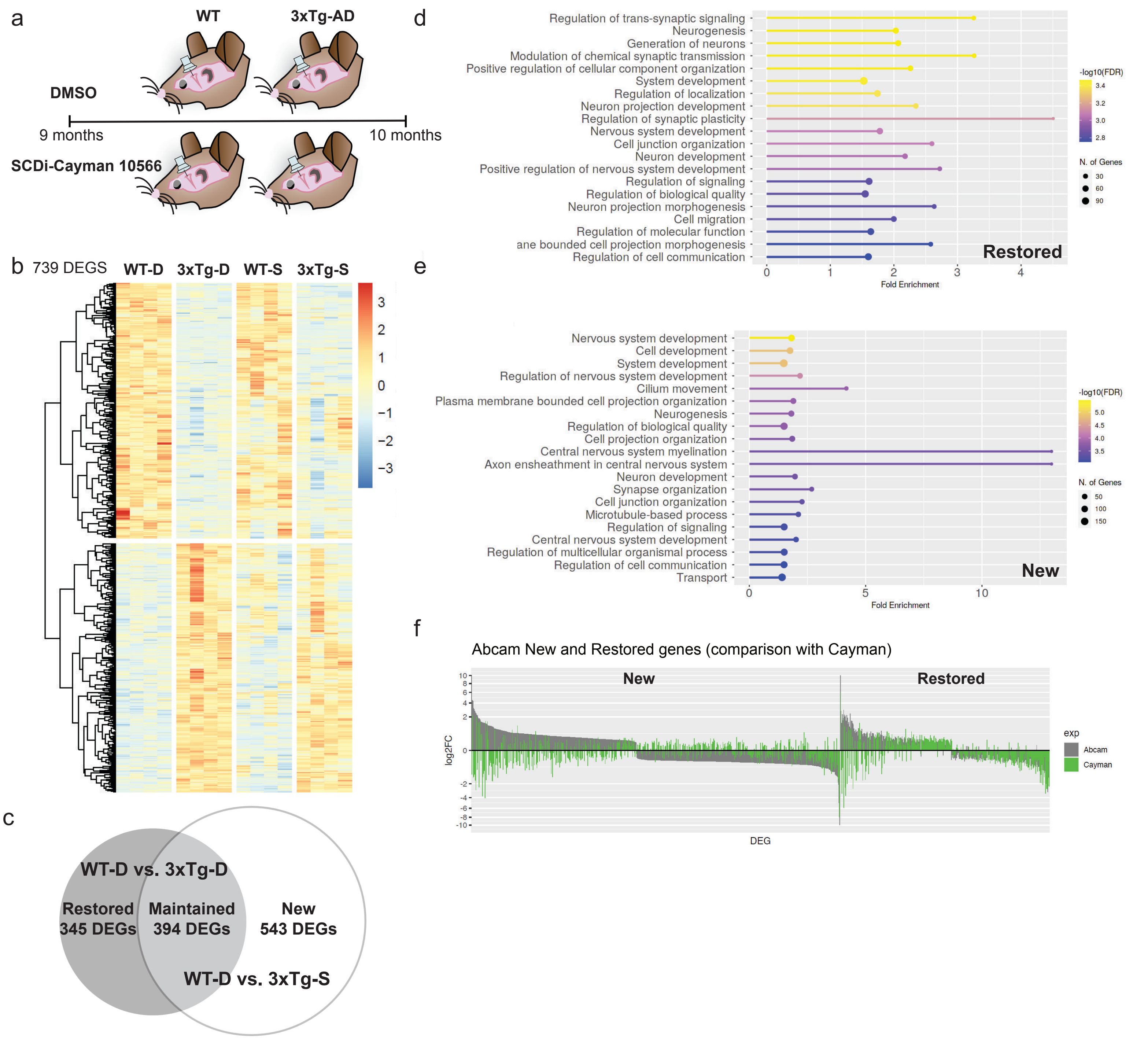

Supplemental Figure 2. Bulk RNA sequencing of the hippocampus following ICV infusion of a second SCDi (CAY10566).

a Timeline of intracerebroventricular infusion experiment on 9-month-old WT and 3xTg mice infused with either vehicle (DMSO) or the SCD inhibitor CAY10566 (SCDi). Hippocampi were extracted after 1 month infusion and processed for whole hippocampus bulk RNAseq.

b Heatmaps of the 739 DEGs ($p \leq 0.01$) between WT-D and 3xTg-D groups, with DEG expression shown across the 4 treatment groups (n=4 mice/group).

c Venn diagram showing the overlap of the WT-D/3xTg-D and WT-D/3xTg-S DEG lists, to identify DEGs that are “Restored” (no longer significant), “Maintained” (still significant), or “New” (newly appearing) after SCDi infusion into 3xTg mice. 345 DEGs were restored by CAY10566, 394 DEGs were maintained, and 543 genes were newly changed.

d,e GO enrichment analysis showing the top 20 most enriched GO Biological Process gene sets (FDR≤0.05) for the “Restored” DEGs (**e**) and “New” DEGs (**f**) in SCDi-infused 3xTg mice. Restored and New DEGs were mainly enriched in GO Biological Process gene sets relating to neural development and synapses. See **Supplemental Data File 2** for complete DEG lists.

f Expression levels (log2FC versus WT-D) of the “New” and “Restored” DEGs from the SCDi ab142089 experiment (grey bars), overlayed with the expression levels (log2FC

versus WT-D) of the same genes from the SCDi CAY10566 experiment (green bars). Note the overlap that is particularly strong for the “Restored” genes.

Related to **Figure 2**.

Supplemental Figure 3

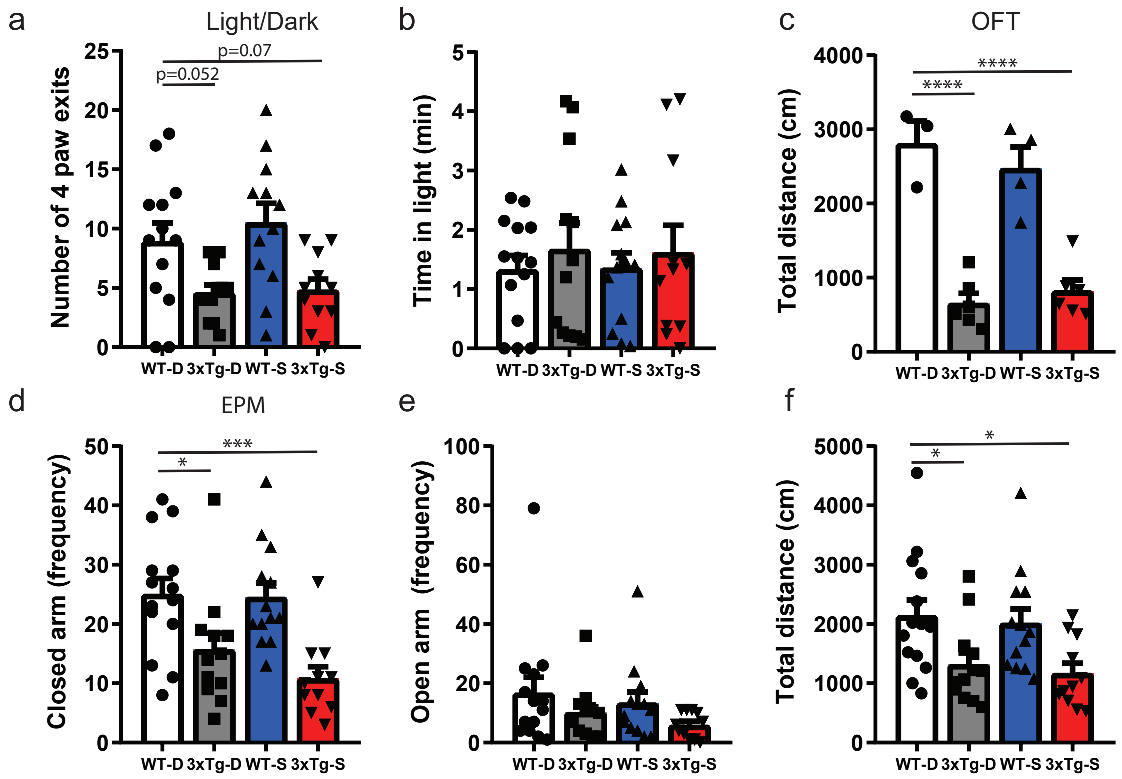

Supplemental Figure 3. Anxiety behaviours are not rescued by 1-month SCDi infusion.

a,b Light/Dark test for WT-D (n=14), 3xTg-D (n=12), WT-S (n=13), 3xTg-S (n=11). Number of 4 paw exits (**a**) and time in the light (**b**). Two-way ANOVA, Dunnett's post-hoc test.

c Open field test on WT-D (n=3), 3xTg-D (n=4), WT-S (n=6), 3xTg-S (n=6). Total distance travelled was significantly decreased between WT-D/3xTg-D (p=0.0001) and WT-D/3xTg-S (p=0.0001). Two-way ANOVA, Dunnett's post-hoc test. ****p≤0.0001.

d-f Elevated plus maze on WT-D (n=13), 3xTg-D (n=12), WT-S (n=12), 3xTg-S (n=11). The frequency in closed arms was significantly decreased between WT-D/3xTg-D (p=0.029) and WT-D/3xTg-S (p=0.0008) (**d**), the frequency in open arms was not significantly different (**e**) and the total distanced travelled was significantly decreased between WT-D/3xTg-D (p=0.038) and WT-D/3xTg-S (p=0.014) (**f**). Two-way ANOVA, Dunnett's post-hoc test. *p≤0.05, ***p≤0.001.

Related to **Figure 3.** Source data are provided as a Source Data file.

Supplemental Figure 4

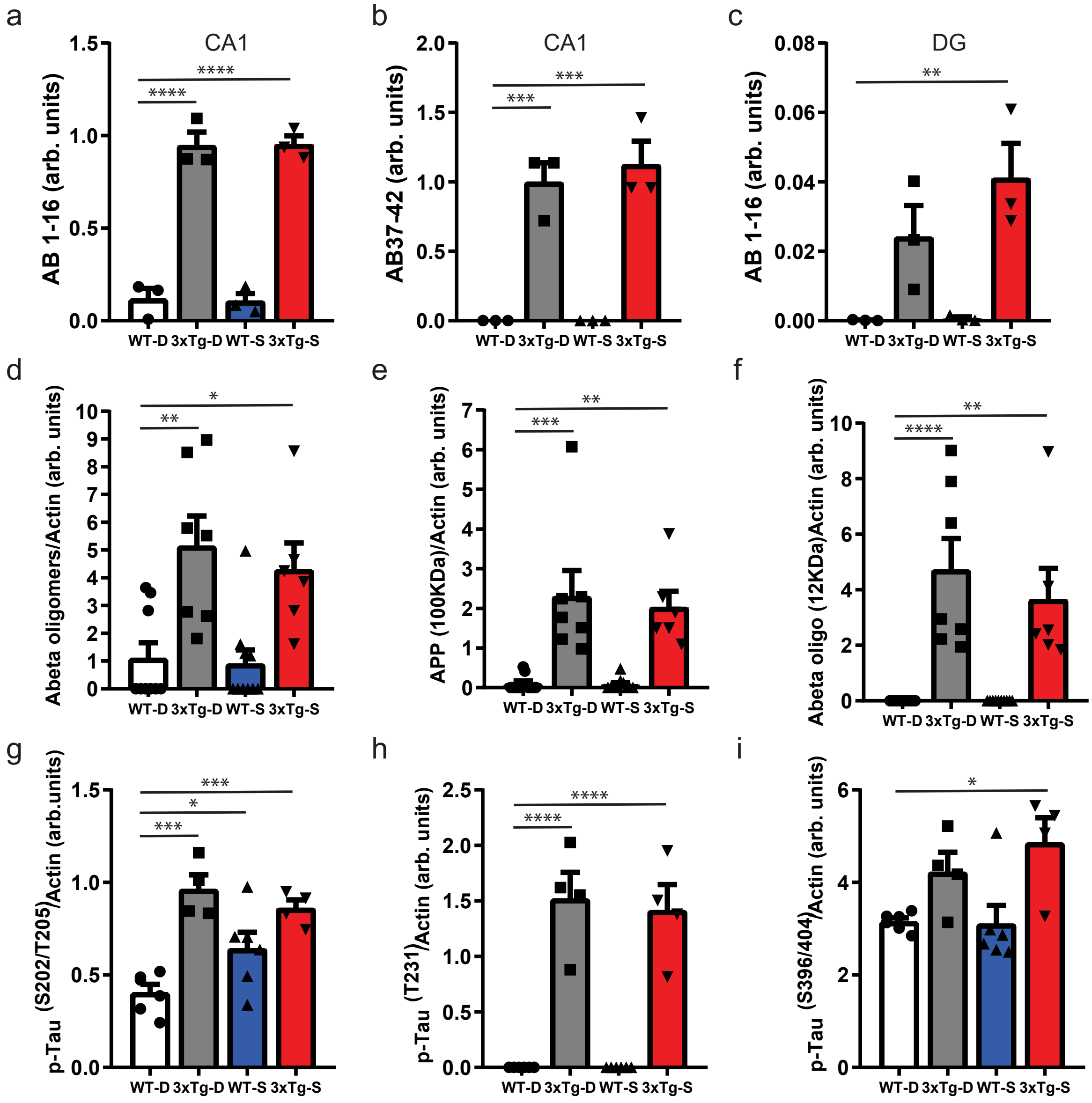

Supplemental Figure 4. Increased amyloid load and tau hyperphosphorylation are not rescued by 1-month SCDi infusion.

a-c Densitometry quantification of immunohistochemistry from WT-D (n=3), 3xTg-D (n=3), WT-S (n=3), 3xTg-S (n=3) showed an increase in two amyloid beta markers between WT-D/3xTg-D (AB1-16 (6E10), p=0.0001) (**a**) (AB37-42, p=0.0005) (**b**) that was unchanged by SCDi (WT-D/3xTg-S, p=0.0001, p=0.0002 respectively) in the CA1. In the DG, AB1-16 (6E10) showed a trend towards significance between WT-D/3xTg-D (p=0.085) and showed significance between (WT-D/3xTg-S, p=0.007) (**c**), while no signal for AB37-42 was observed. Two-way ANOVA, Dunnett's post-hoc test. Arbitrary units (arb. units). **p≤0.01, ***p≤0.001, ****p≤0.0001.

d-f Western blot quantifications of AB1-16 (6E10) (**d,e**) or AB37-42 (**f**) in the hippocampus in WT-D (n=9), 3xTg-D (n=10), WT-S (n=7), 3xTg-S (n=6). Quantification of the 12 kDa band (oligomeric amyloid beta) using AB1-16 (6E10) showed a significant increase between WT-D/3xTg-D (p=0.002) and WT-D/3xTg-S (p=0.020) (**d**) as did quantification of the 12 kDa band (oligomeric amyloid beta) using AB37-42 WT-D/3xTg-D (p=0.0001) and WT-D/3xTg-S (p=0.002) (**e**). An increase in total APP was also observed between WT-D/3xTg-D (p=0.002) and WT-D/3xTg-S (p=0.001) (**f**). Two-way ANOVA, Dunnett's post-hoc test. All bands were normalized to Actin. *p≤0.05, **p≤0.01, ***p≤0.001, ****p≤0.0001.

g-i Quantification of three stages of Tau phosphorylation in WT-D (n=6), 3xTg-D (n=4), WT-S (n=6), 3xTg-S (n=4). Early p-Tau$^{(S202/T205)}$ was increased between WT-D/3xTg-D

(p=0.0001) and WT-D/3xTg-S (p=0.001) (**g**), mid p-Tau$^{(T231)}$ was increased between WT-D/3xTg-D (p=0.0001) and WT-D/3xTg-S (p=0.0001) (**h**) and late p-Tau$^{(S396/404)}$ was only significantly different between WT-D/3xTg-S (p=0.014) (**i**). Two-way ANOVA, Dunnett's post-hoc test. All bands were normalized to Actin. *p≤0.05, ***p≤0.001, ****p≤0.0001. Error bars represent mean +/- SEM. Arbitrary units (arb. units).

Related to **Figure 3.** Source data are provided as a Source Data file.

Supplemental Figure 5

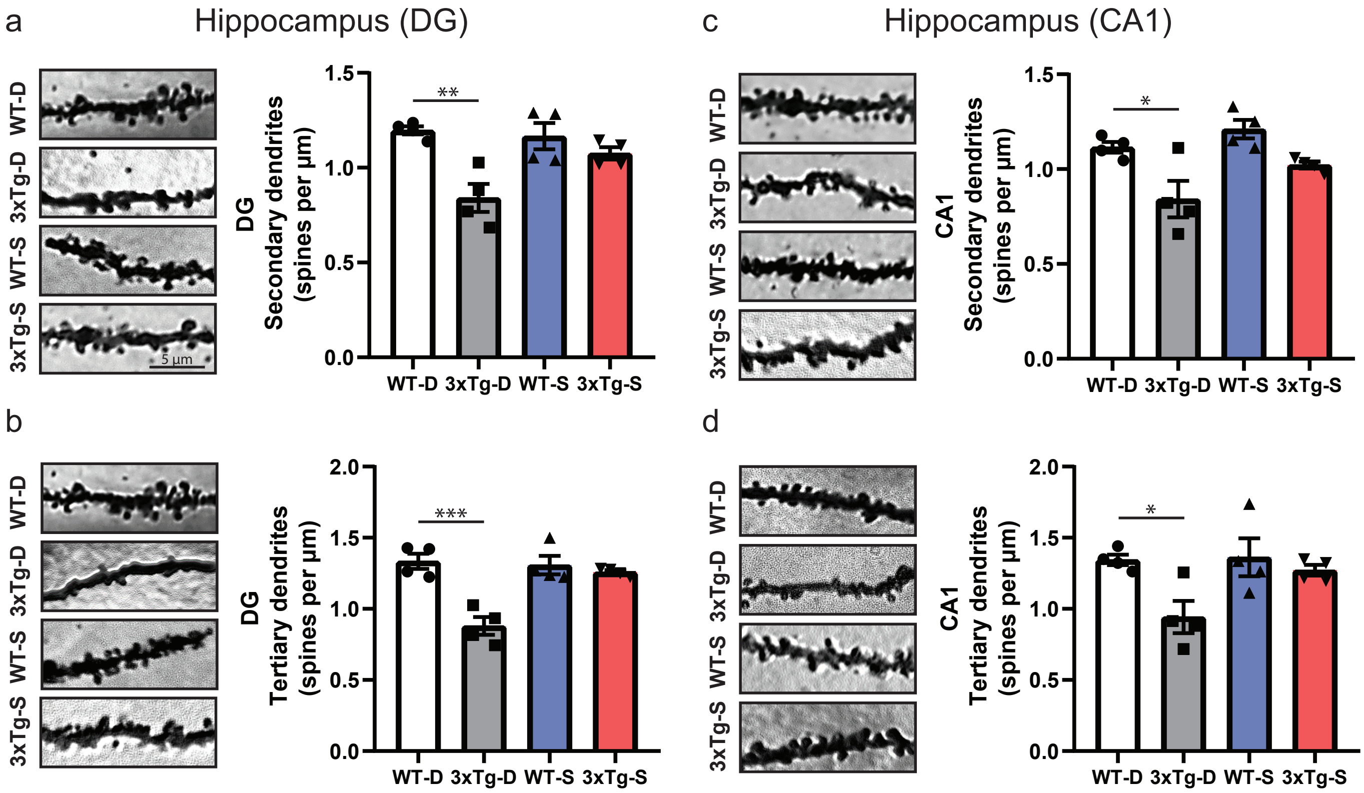

Supplemental Figure 5. A second SCDi, CAY10566, also rescues dendritic spine loss in 3xTg hippocampal neurons.

a-d Golgi staining in the hippocampus. Quantification of the number of spines on dentate gyrus (DG) neurons (average of 8 neurons from each of 4 animals/group) (**a**,**b**). DG secondary dendrites showed a significant decrease between WT-D/3xTg-D (p=0.0015) that was no longer present following SCDi (WT-D/3xTg-S, p=0.307) (**a**), tertiary dendrites showed a similarly significant decrease between WT-D/3xTg-D (p=0.0002) that was no longer present following SCDi (WT-D/3xTg-S, p=0.628) (**b**), 2-way ANOVA with Dunnett's post-hoc test. Quantification of the number of spines on CA1 neurons (average of 8 neurons from each of 4 animals/group) (**c**,**d**). CA1 secondary dendrites showed a significant decrease between WT-D/3xTg-D (p=0.013) that was no longer present following SCDi (WT-D/3xTg-S, p=0.528) (**c**), tertiary dendrites showed a similarly significant decrease between WT-D/3xTg-D (p=0.024) that was no longer present following SCDi (WT-D/3xTg-S, p=0.898) (**d**), 2-way ANOVA, Dunnett's post-hoc test. *p≤0.05, **p≤0.01, ***p≤0.001. Error bars represent mean +/- SEM. Scale bar, 5um.

Related to **Figure 4**. Source data are provided as a Source Data file.

Supplemental Figure 6

a b

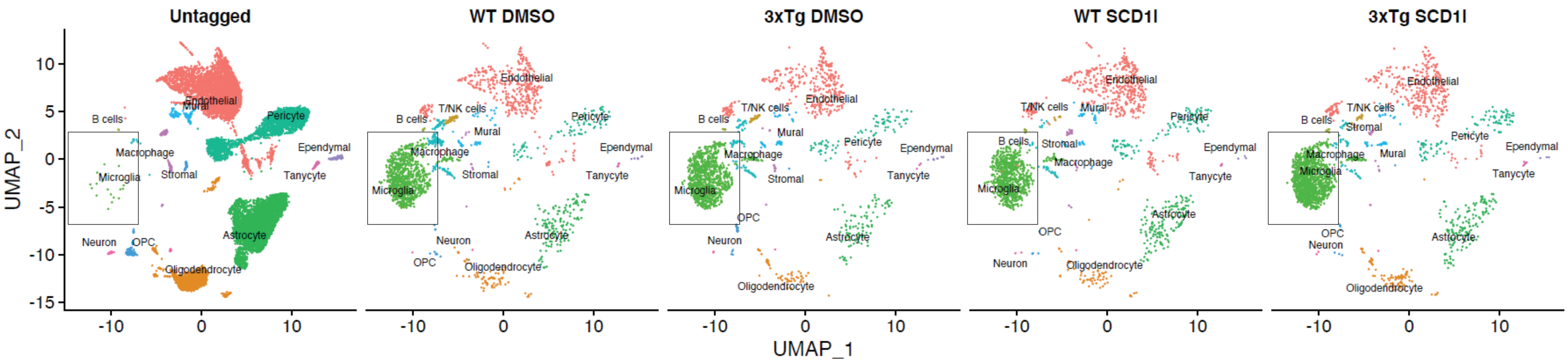

c

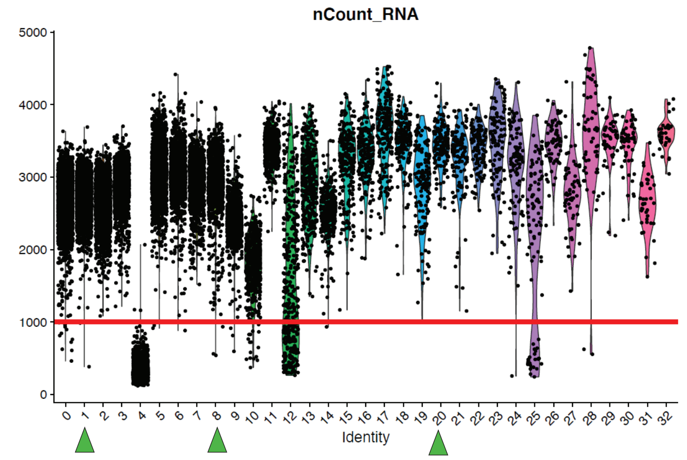

d

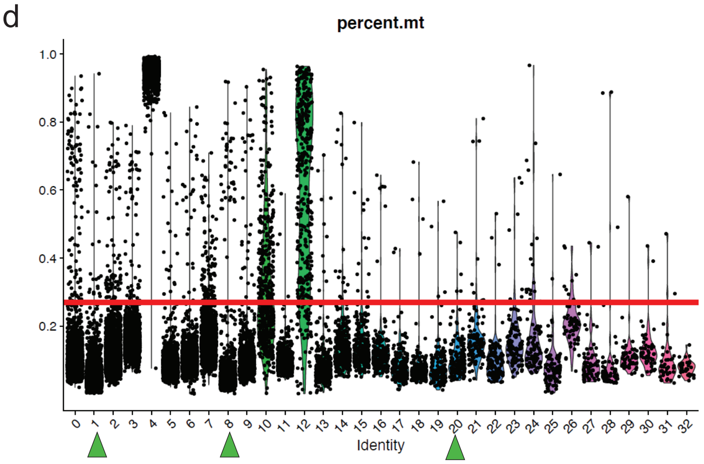

e

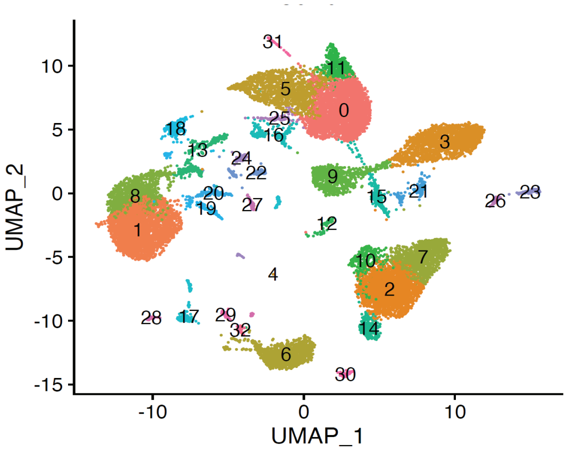

f

	WT DMSO	3xTg DMSO	WT SCD1i	3xTg SCD1i	Untagged
Endothelial	427	348	456	366	3567
Oligodendrocyte	75	57	63	90	1121
T/NK cells	55	30	7	22	0
B cells	6	8	7	6	2
Microglia	843	921	780	1203	19
Astrocyte	136	93	150	147	3293
Pericyte	84	82	105	103	2111
Macrophage	170	87	36	50	11
Mural	41	51	44	46	113
OPC	11	23	6	9	136
Ependymal	6	8	3	3	81
Stromal	9	13	29	15	79
Tanycyte	4	3	3	6	44
Neuron	2	3	3	3	49
Total	1869	1727	1692	2069	10626

g

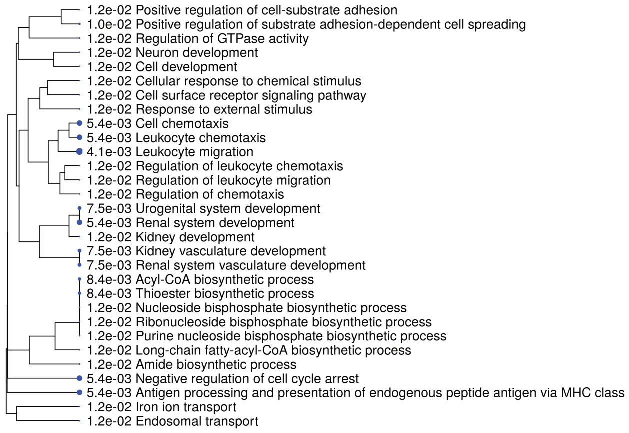

Supplemental Figure 6. Single cell RNA sequencing of the WT and 3xTg hippocampus.

a,b UMAP plots of untagged (**a**) and CD45-tagged (**b**) cells from hippocampi of ICV pump mice (pooled n=4 per group) showing cell type annotations.

c,d Single cell RNA sequencing nCount RNA (**c**) and mitochondrial(mt) RNA percent (%) (**d**) that were used to threshold out dead/dying cells. Green arrowheads identify the cell clusters (1,8,20) annotated as microglia.

e UMAP of cell clusters from all cells that passed QC threshold (**e**).

f Table of cell numbers of each annotated cell type.

g GO enrichment analysis of combined Restored and New microglial DEG list (FDR≤0.05).

Related to **Figures 5 and 6**.

Supplemental Figure 7

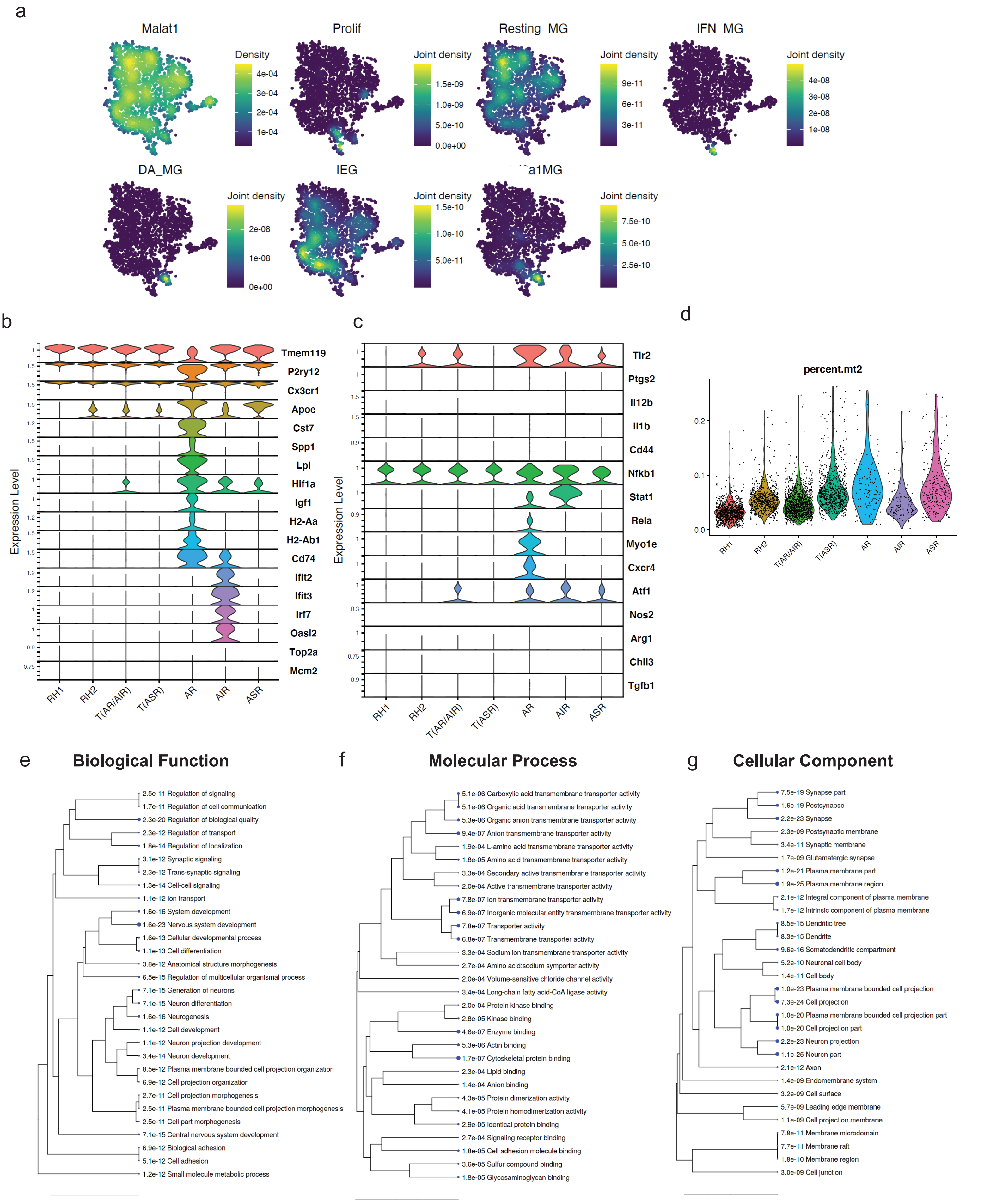

Supplemental Figure 7. Cell type annotations for single cell clusters.

a Density plots of expression of microglia subtype markers from Friedman et al. [35] in our dataset.

b Violin plots of microglia subtype marker expression from Sala Frigerio et al. [34] in our dataset.

c Violin plots of common microglia subtype markers in our dataset.

d Violin plots of mitochondrial content (%) across microglia subclusters.

e-g GO enrichment analysis (FDR≤0.05) of DEGs defining the subcluster 4 (ASR) microglia subtype. **e** Biological function, **f** Molecular process, **g** Cellular component.

Related to **Figure 6**.

Supplemental Figure 8

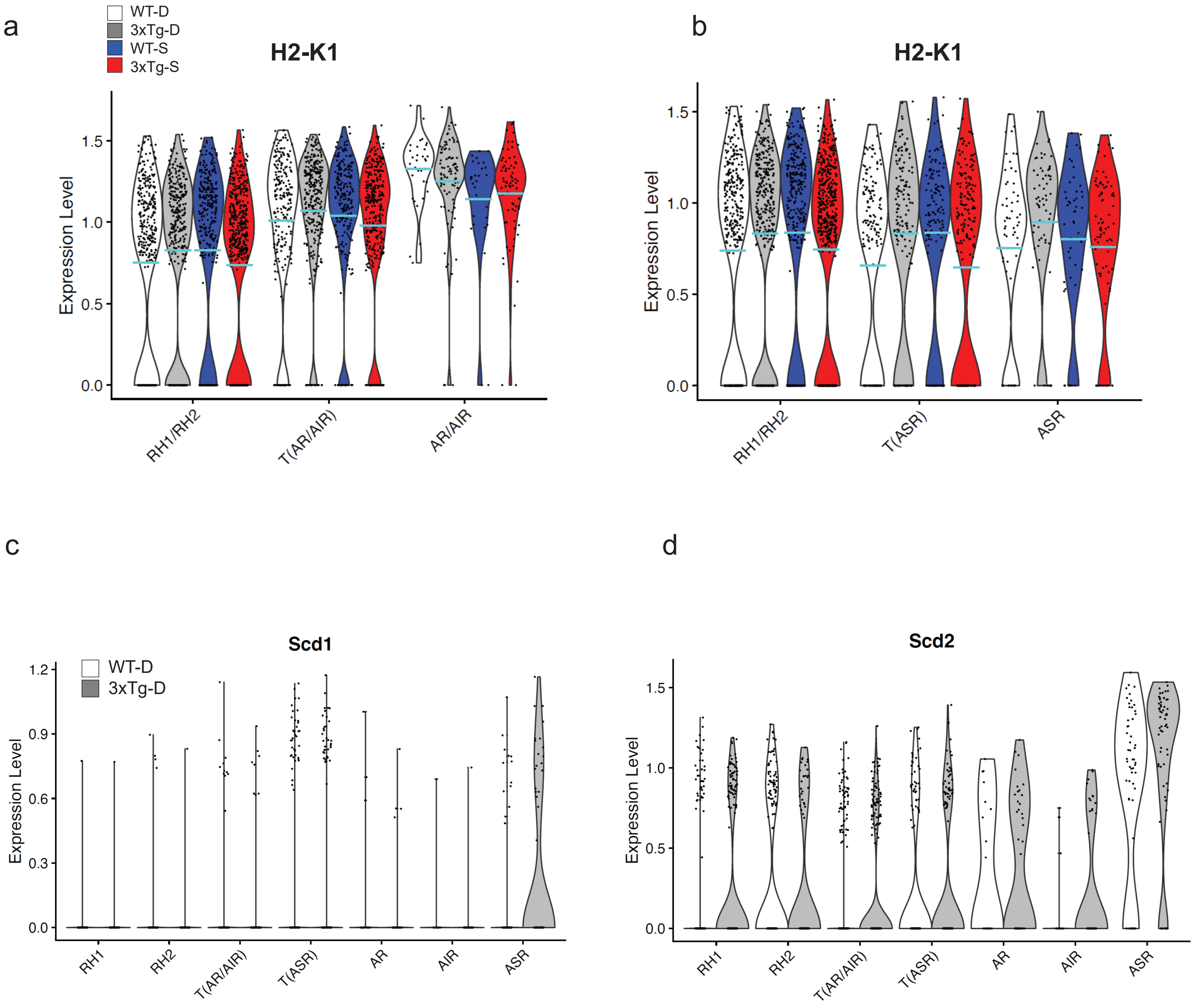

Supplemental Figure 8. Expression of Scd and MHC-I genes in microglia subclusters.

a,b Violin plots of expression levels of H2-K1 in the AR/AIR lineage (**a**) and ASR lineage (**b**) in each experimental group. Blue line identifies the average expression level.

c,d Violin plots of expression level of Scd1 (**c**) and Scd2 (**d**) in microglia subtypes of WT-D and 3xTg-D experimental groups.

Related to **Figure 6**.

Supplemental Data File 1. 8M whole hippocampus RNAseq DEG list associated to **Figure 1**.

Supplemental Data File 2. WT-D, 3xTg-D WT-S, 3xTg-S whole hippocampus RNAseq DEG lists associated to **Figure 2**.

Supplemental Data File 3. Single cell RNAseq DEG list associated to **Figure 3**.

Supplemental Table 1. qPCR primer list
