# Supplementary material for: Stearoyl-CoA Desaturase inhibition reverses immune, synaptic and cognitive impairments in an Alzheimer’s disease mouse model
Source: Nat Commun. 2022 Apr 20;13:2061. doi: 10.1038/s41467-022-29506-y (PMC9021296; doi:10.1038/s41467-022-29506-y)
Supplement: Supplementary file 2 — Reporting Summary [file 41467_2022_29506_MOESM2_ESM.pdf]

nature research

Corresponding author(s): Karl Fernandes

Last updated by author(s): Mar 1, 2022

Reporting Summary

Nature Research wishes to improve the reproducibility of the work that we publish. This form provides structure for consistency and transparency in reporting. For further information on Nature Research policies, see our Editorial Policies and the Editorial Policy Checklist.

Statistics

For all statistical analyses, confirm that the following items are present in the figure legend, table legend, main text, or Methods section.

n/a	Confirmed	
☐	☒	The exact sample size (n) for each experimental group/condition, given as a discrete number and unit of measurement
☐	☒	A statement on whether measurements were taken from distinct samples or whether the same sample was measured repeatedly
☐	☒	The statistical test(s) used AND whether they are one- or two-sided<br>*Only common tests should be described solely by name; describe more complex techniques in the Methods section.*
☐	☒	A description of all covariates tested
☐	☒	A description of any assumptions or corrections, such as tests of normality and adjustment for multiple comparisons
☐	☒	A full description of the statistical parameters including central tendency (e.g. means) or other basic estimates (e.g. regression coefficient) AND variation (e.g. standard deviation) or associated estimates of uncertainty (e.g. confidence intervals)
☐	☒	For null hypothesis testing, the test statistic (e.g. F, t, r) with confidence intervals, effect sizes, degrees of freedom and P value noted<br>*Give P values as exact values whenever suitable.*
☒	☐	For Bayesian analysis, information on the choice of priors and Markov chain Monte Carlo settings
☐	☒	For hierarchical and complex designs, identification of the appropriate level for tests and full reporting of outcomes
☒	☐	Estimates of effect sizes (e.g. Cohen's d, Pearson's r), indicating how they were calculated

Our web collection on statistics for biologists contains articles on many of the points above.

Software and code

Policy information about availability of computer code

Data collection	BD Rhapsody Express for single cell capture (BD Rhapsody scanner software, BD Bioscience)<br>Behaviour measurement acquisition: Ethovision XT 15<br>Western blot acquisition: Chemidoc Image lab software version 4.1 (Bio-Rad)
Data analysis	For bulk RNA seq:<br>Alignment and count matrix generation for bulk RNAseq: STAR 2.5.1b<br>Bulk RNAseq normalization and DE analysis and visualization: R 4.0.3, TCC 1.24.0, pheatmap 1.0.12<br>GO enrichment analysis: ShinyGO v0.61,ShinyGO v0.741<br>GSEA analysis: GSEA v4.0.3<br><br>For single cell RNA seq:<br><br>Alignment, demultiplexing and count matrix generation for single cell RNAseq data: BD RhapsodyTM WTA Analysis Pipeline CWL v1.0 on Seven Bridges Platform<br>Preprocessing and clustering scRNAseq: scanpy 1.4.3 on GenAP platform (using Galaxy 20.01)<br>Downstream scRNAseq annotation and analysis: R 4.0.3, Seurat 4.1.0, ScibetR 0.1.0, scMCA 0.2.0, FindMarkers,<br>Trajectory inference: dyno 0.1.2<br>GO enrichment analysis: ShinyGO v0.61,ShinyGO v0.741<br>GSEA analysis: GSEA v4.0.3<br>To cross reference DEG lists: molbiotools (https://molbiotools.com/listcompare.php).<br>For Behavioral analysis: Ethovision XT 15

For dendritic analysis of Golgi stained neurons: Fiji v1.52p

For amyloid and Tau IHC quantification: Fiji v1.52p

For PSD95/Iba1 analysis:Fiji v1.52p

For amyloid and Tau Western blot quantification: Image Lab software version 4.1 (Bio-rad)

Statistics: GraphPad Prism, Version 8

OpenLab ChemStation, Version C.01.10

For manuscripts utilizing custom algorithms or software that are central to the research but not yet described in published literature, software must be made available to editors and reviewers. We strongly encourage code deposition in a community repository (e.g. GitHub). See the Nature Research guidelines for submitting code & software for further information.

Data

Policy information about availability of data

All manuscripts must include a data availability statement. This statement should provide the following information, where applicable:

- Accession codes, unique identifiers, or web links for publicly available datasets
- A list of figures that have associated raw data
- A description of any restrictions on data availability

The datasets generated during the current study are available at GEO, accession number GSE167605
Figures 1,2,5,6 have associated raw data.
There are no restriction on data availability

Field-specific reporting

Please select the one below that is the best fit for your research. If you are not sure, read the appropriate sections before making your selection.

☒ Life sciences ☐ Behavioural & social sciences ☐ Ecological, evolutionary & environmental sciences

For a reference copy of the document with all sections, see nature.com/documents/nr-reporting-summary-flat.pdf

Life sciences study design

All studies must disclose on these points even when the disclosure is negative.

Sample size	Samples sizes were determined by previous experience taking the number of variables in the experiment under consideration. For example when behaviour was to be performed, approximately 10 mice per group was chosen in advance based on similar prior publications (Vander Jeugd et al 2018). While, if cellular counting or culture was required 3-5 mice per group was deemed to be sufficient based on our prior publications using similar methods (Hamilton et al 2010, 2015).<br>Figure 1/Figure 2: For whole hippocampus (bulk) RNA sequencing experiments, we used an N of 3 biological replicates per condition.<br>Figure 3: For behavioral studies, MWM: we used N=11-14 mice per treatment group. For immunohistochemistry for markers of neurogenesis we used N of 3-5 animals per treatment group (Figure 3). For EPM/LD: 11-14 mice per treatment group, were used, for OFT: 3-6 mice per treatment group were used (supplemental figure 3). Immunohistochemistry for amyloid was performed on N=3 animals. Western blotting for amyloid was performed on N 6-10 animals per treatment group, Western blotting for tau antibodies was performed on N= 4-6 animals per treatment group (supplemental figure 4).<br>Figure 4: For Golgi staining, we used an N of 4 biological replicates per condition and performed spine and dendritic quantifications from approximately 5-14 labeled neurons from each N. (the same analysis was performed for Cayman SCDi (supplemental Fig 5). For Nissl, we used 4 sections from N of 4-6 animals.<br>Figure 5: For PSD95/Iba-1 quantification we quantified 5-14 microglial cells per animal from 4-5 animals per treatment group, for single cell RNA sequencing studies, we analyzed a total of 1692-2069 single cells per experimental group, with cells from each experimental group derived from an N=4 pooled animals. For microglia cultures, WT n=8, animals/treatment group, Scd1-KO n=3 animals/treatment group.<br>Figure 6: For single cell RNA sequencing studies, we analyzed a total of 1692-2069 single cells per experimental group, with cells from each experimental group derived from an N=4 pooled animals.
Data exclusions	No data was excluded from the analysis
Replication	Figure 1: Bulk RNAseq (non-operated mice) performed once with N=3 per group.<br>Figure 2: Bulk RNAseq (ICV infused mice) performed once with N=3 per group. Replicated using Cay SCDi.<br>Figure 3: For behavioral studies, MWM: 5 separate cohorts (8 mice per cohort, with all experimental groups represented in each cohort). EPM/LD: Mice were processed in 2 separate cohorts, with all experimental groups represented in each cohort). OFT: Mice processed in 1 cohort. Staining for markers of neurogenesis and amyloid was performed once on 3 sections per animal., Nissl staining was performed once, on 4 sections per animal.<br>Figure 4: Golgi staining was performed once with N=4 mice/group and on multiple sections per animal. This was replicated using a second SCD inhibitor, Cayman SCDi.

Figure 5/6: Staining for PSD95/Iba-1, was performed once on 2 sections per animal. Single cell RNAseq on a pool of 4 mice per treatment group was performed once. Microglia cultures were performed in three batches, with WT and KO's present in each experiments, 1 well per condition.

Randomization

When treatments were administered, animals were divided into groups randomly.

Blinding

Quantifications of spines, dendrites, PSD95/Iba1, neurogenesis markers, amyloid and tau were performed by a blinded experimenter. For microglia culturing , the experimenter was blinded until genotypes were determined before treatment. All bioinformatic analysis of RNA sequencing data was performed by collaborator that was not part of the study. For behavioural tests the experimenter was blind to the animals experimental group and strain during data acquisition and analysis.

Reporting for specific materials, systems and methods

We require information from authors about some types of materials, experimental systems and methods used in many studies. Here, indicate whether each material, system or method listed is relevant to your study. If you are not sure if a list item applies to your research, read the appropriate section before selecting a response.

Materials & experimental systems

n/a	Involved in the study
☐	☒ Antibodies
☒	☐ Eukaryotic cell lines
☒	☐ Palaeontology and archaeology
☐	☒ Animals and other organisms
☒	☐ Human research participants
☒	☐ Clinical data
☒	☐ Dual use research of concern

Methods

n/a	Involved in the study
☒	☐ ChIP-seq
☒	☐ Flow cytometry
☒	☐ MRI-based neuroimaging

Antibodies

Antibodies used

Mouse anti-human Ki67 (cat#556003, BD Biosciences, 1:100), rabbit anti-GFAP (cat# Z0334, Dako Diagnostic/Agilent, 1:1000), guinea-pig anti-DCX (cat# AB2253, Millipore, 1:3000), rabbit anti-Abeta 37-42 D54D2 XP (cat#8 243, NEB, 1:100), mouse Abeta 1-16 (cat# 803001, Covance, 6E10, 1:200), mouse PHF1 (p-Tau(S396/404))(Peter Davis, 1:100), mouse AT8 (p-Tau(S202/T205))(cat# MN1020, Thermo, 1:100), mouse AT180 (p-Tau(T231))(cat# MN1040, Thermo, 1:100), PSD-95 clone 6G6-1C9(cat# MAB1596, Millipore, 1:160), Iba-1 (cat# 019-19741, Wako, 1:400). HRP Goat anti rabbit (cat#111-035-144, Jackson Immuno Research, 1:5000), HRP Goat anti mouse (cat#170-6516, Bio-Rad, 1:5000), Alexa 488 Donkey anti-rabbit (H+L) (cat#A21206, Invitrogen, 1:1000), Alexa 555 Goat anti-guinea pig (H+L) (cat#A21435, Invitrogen, 1:1000), Alexa 647 Donkey anti-mouse (H+L) (cat#A31570, Invitrogen, 1:1000). Biotin Goat anti mouse (H+L) (cat#115-065-146, Jackson Immuno Research, 1:1000),Biotin Goat anti rabbit (H+L) (cat#111-065-144, Jackson Immuno Research, 1:1000)

Validation

Ki67: The B56 monoclonal antibody specifically binds to the Ki-67 antigen that is expressed in the nucleus of cycling cells (G1, S, G2, M cell cycle phases) as stated by BD biosciences. (Has been used in our prior publication Hamilton et al 2015).
GFAP: In the central nervous system Z0334 labels astrocytes and some groups of ependymal cells as stated by Agilent technologues. (Has been used in our prior publication Hamilton et al 2015).
DCX: Anti-Doublecortin Antibody, Cat. No. AB2253, is a highly specific guinea pig polyclonal antibody that targets Doublecortin as stated by Millipore. (Has been used in our prior publication Hamilton et al 2015).
Abeta37-42: β-Amyloid (D54D2) XP® Rabbit mAb recognizes endogenous levels of total β-amyloid peptide (Aβ). The antibody detects several isoforms of Aβ, such as Aβ-37, Aβ-38, Aβ-39, Aβ-40, and Aβ-42. This product detects transgenically expressed human APP in mouse models according to Cell Signalling Technologies. ex: Western Blot publication(Kaku et al 2021)
Abeta 1-16: This antibody is reactive to amino acid residue 1-16 of beta amyloid. The epitope lies within amino acids 3-8 of beta amyloid (EFRHDS) according to Biolegend. ex: Western Blot publication, Miyamoto et al. 2016)
PHF1: PHF1 is a mouse monoclonal antibody specific towards phosphorylation sites S396 and S404 in tau (Otvos L. et al 1994).
AT8:MN1020 targets PHF-tau (Ser202/Thr205)a in ELISA, IF, IHC(P), and WB applications and shows reactivity with Human samples according to Thermo Fisher.
AT180:MN1040 targets PHF-tau (Thr231) in IF, IHC, and WB applications and shows reactivity with human and rat samples according to Thermo Fisher.
The tau antibodies (PHF1, AT8, AT180) used in the current study have been validated for specificity ex: (https://www.biorxiv.org/content/10.1101/612911v1.full.pdf).
PSD95: Anti-Post Synaptic Density Protein 95 Antibody, clone 6G6-1C9 detects level of Post Synaptic Density Protein 95 & has been published & validated for use in IC, IH & WB according to Millipore). ex: Immuno fluorescence publication: Grubman et al. 2021).
Iba-1: Synthetic peptide C-terminal of Iba1, Reactive with human, mouse and rat Iba1 in immunohistochemistry according to Wako. (Has been used in our prior publication Hamilton et al 2015).

All antibodies have been tested for specificity in our lab by omitting either the primary or secondary antibodies and only antibodies that show specific signal under these circumstances were used in this study.

Animals and other organisms

Policy information about studies involving animals; ARRIVE guidelines recommended for reporting animal research

Laboratory animals	The animals used in the current study were female 3xTg-AD mice (Jackson Laboratory MMRRC stock #: 034830) and their WT strain controls (Jackson Laboratory stock no: 101045). For experiments in Figure 1 the mice were sacrificed at 8 months of age, for the remaining experiments the mice were sacrificed at 9-10 months of age. WT/3xTg mice were housed in 12h reverse cycle (Lights OFF:10am ON :10pm), at 22C, 50% humidity. For Fig 5k-n, Scd1 knockout (Scd1-KO) and their WT litter mates were used at postnatal day 1. SCD-WT/SCD-KO mice were housed in 12h regular cycle (Lights ON: 7am OFF: 7pm), at 26 C. 50% humidity .
Wild animals	This study did not involve wild animals
Field-collected samples	This study did not involve field-collected samples
Ethics oversight	Experiments were approved by the Institutional Animal Care Committee of the Centre de Recherche du Centre Hospitalier de l'Université de Montréal (CRCHUM) or Université de Quebec à Montreal (UQAM) following the Canadian Council of Animal care guidelines.

Note that full information on the approval of the study protocol must also be provided in the manuscript.
