# Supplementary material for: Stearoyl-CoA Desaturase inhibition reverses immune, synaptic and cognitive impairments in an Alzheimer’s disease mouse model
Source: Nat Commun. 2022 Apr 20;13:2061. doi: 10.1038/s41467-022-29506-y (PMC9021296; doi:10.1038/s41467-022-29506-y)
Supplement: Supplementary file 4 — Description of Additional Supplementary Files [file 41467_2022_29506_MOESM4_ESM.pdf]

Title: Supplementary Data File 1.
Description: 8M whole hippocampus RNAseq DEG list associated to Figure 1.

Title: Supplementary Data File 2.
Description: WT-D, 3xTg-D WT-S, 3xTg-S whole hippocampus RNAseq DEG lists associated to Figure 2.

Title: Supplementary Data File 3.
Description: Single cell RNAseq DEG list associated to Figure 3.
